# Chemical Composition, Enantiomeric Distribution, and Physical Properties of the Fruit Essential Oil from *Zanthoxylum lepidopteriphilum* (Reynel) Rutaceae from Ecuador

**DOI:** 10.3390/plants13202834

**Published:** 2024-10-10

**Authors:** Vladimir Morocho, Yolanda Aguilar, Claudia Cruz, Nixon Cumbicus, Jose Miguel Andrade, Mayra Montalvan

**Affiliations:** 1 Departamento de Química, Universidad Técnica Particular de Loja, Loja 110150, Ecuador; ymaguilar6@utpl.edu.ec (Y.A.); ctcruz1@utpl.edu.ec (C.C.); jmandrade@utpl.edu.ec (J.M.A.); msmontalvan@utpl.edu.ec (M.M.); 2 Departamento de Ciencias Biológicas y Agropecuarias, Universidad Técnica Particular de Loja (UTPL), San Cayetano s/n, Loja 1101608, Ecuador; nlcumbicus@utpl.edu.ec; 3 Instituto Universitario de Bio-Orgánica Antonio González (IUBO AG), Universidad de La Laguna (ULL), Avda. Astrofísico F. Sánchez 2, 38206 La Laguna, Tenerife, Spain

**Keywords:** *Zanthoxylum lepidopteriphilum*, α-thujone, β-thujone

## Abstract

The essential oil was obtained by steam distillation, using a Clevenger apparatus, from the pericarp of the fruit of *Zanthoxylum lepidopteriphilum* from Ecuador. The qualitative and quantitative analyses were performed by gas chromatography coupled with mass spectrometry (GC-MS) and flame ionization detection (GC-FID) on two capillary columns with non-polar DB-5ms and a polar HP-INNOWax stationary phase. Thirty-three components were identified, accounting for 99.62% and 99.30% total essential oil. The essential oil was dominated by oxygenated monoterpenes (90.21–89.21%), respectively. The main constituents of the essential oil were α-thujone (70.26–70.38%), β-thujone (10.78–10.90%), terpinen-4-ol (4.15–4.06%), and sabinene (3.60–4.02%). Enantioselective analysis by GC was realized on a β-cyclodextrin-based chiral column (2,3-diethyl-6-tert-butyldimethylsilyl-β-cyclodextrin) in this analysis, determining three couples of enantiomers, which exhibited the compound (*1R*,*4S*,*5S*)-(+)-α-thujone with an enantiomeric excess of 84.40%.

## 1. Introduction

Since ancient times, plants have been used to treat, prevent, and cure diseases. In recent years, the use of plants with medicinal potential has increased significantly, driving research focused on the characterization, identification, and isolation of new natural products with therapeutic properties. Among these natural products, essential oils, commonly known as flavorings or essences, have found wide application in various fields [1]. 

Plants play a therapeutic and pharmaceutical role in protecting human beings from the effects of diseases and other complications, thus considered to have a significant role in the healthcare system. A progressive increase in medicinal plant usage has been recorded continuously, both for traditional users and the pharmaceutical industry [2]. 

Members of the Rutaceae family are characterized by distinct oil glands in their aromatic leaves, and while their flowers are predominantly perfect, they can occasionally be unisexual. This family is well-known for its rich phytochemical composition and associated medicinal properties [3].

*Zanthoxylum* is the largest and most widespread genus in the family Rutaceae [4]. It is distributed worldwide in the tropics and in temperate zones of the new and old worlds [5] and is composed of 666 species. The genus ranges from shrubs to tall trees, with most of the *Zanthoxylum* species being timber trees [6].

It has great importance due to its ethnobotanics, phytochemistry, and biological activity, and it is a promising source of various secondary metabolites, including benzophenanthridine alkaloids, in addition to accumulating volatile oils in its leaves, fruits, and inflorescences [7,8]. 

The richness of *Zanthoxylum* is found in essential oils, mainly from leaves, flowers, seeds, and fruits [9]. Most of the plants are dioic; in the fruits of different species, it has been found that these oils are complex mixes of terpene compounds [10]. 

In Ecuador, the genus *Zanthoxylum* comprises around 20 species [11,12,13]. Numerous studies at the agronomic, economic, and market level, as well as a detailed investigation of the chemical, physical, and biological composition of the oils obtained from the cultivated, introduced, or native plants, allow the development of the essential oils industry [14]. 

Approximately 90% of global essential oil (EO) production is consumed by the flavor and fragrance industries, primarily in the form of perfumes, flavorings, and condiments. [15,16]. *Zanthoxylum lepidopteriphilum* is a native shrub found in the Andean region of Ecuador. It is located in the Loja Province at 2220 m a.s.l. Previous reports have not been found regarding *Z. lepidopteriphilum*. Therefore, this study aims to investigate the chemical composition and enantiomeric profile of the essential oil from *Z. lepidopteriphilum*, providing valuable insights into its potential as a source for novel pharmaceutical, cosmetic, and food products.

## 2. Results

### 2.1. Obtention the Essential Oil and Physical Properties

The essential oil of *Z. lepidopteriphilum* fruits was obtained by steam distillation for 4 h. The essential oil yield was 1.38% ± 0.29 (*w*/*w*). Three physical properties were determined: refractive index 1.45 ± 0.00008, relative density 0.87 ± 0.001 g/mL, and optical rotation, with an α = +1.86 in CH_2_Cl_2_, *c* = 0.1.

### 2.2. Chemical Composition of Essential Oil 

**Figure 1:**
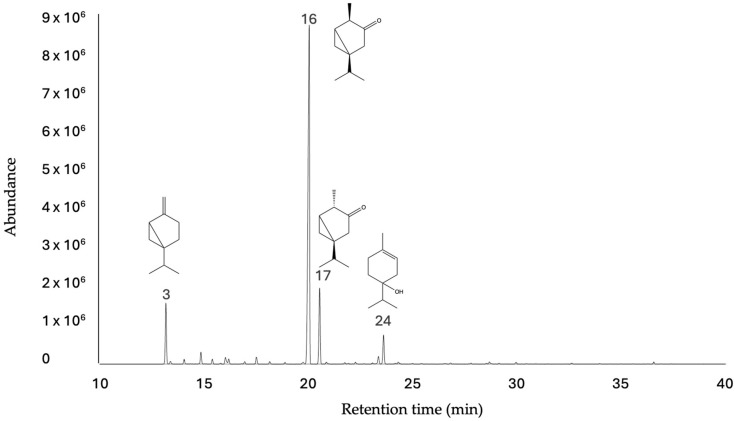
Chromatogram of essential oil from *Zanthoxylum lepidopteriphilum* fruits in DB-5ms column.

Thirty-three compounds were identified by GC-MS and GC-FID in the essential oil of *Z. lepidopteriphilum* fruits, which represented 99.62% (DB-5ms) and 99.30% (HP-INNOWax) of the total composition of essential oil (Figure 1). The main compounds were the monoterpenes α-thujone (70.26%) and β-thujone, (10.78%). The major constituents found in the essential oil were monoterpenes (76.63%), predominately composed of oxygenated monoterpenes (90.21%), and monoterpene hydrocarbons (8.50%). The results of GC-MS and GC-FID analyses are reported in Table 1. 

### 2.3. Enantioselective Analysis

Analysis by GC-MS with two columns MEGA-DEX DET Beta (2,3-diethyl-6-tert-butyldimethylsilyl-β-cyclodextrin) and MEGA-DEX DAC Beta (2,3-diacetyl-6-tert-butyldimethylsilyl-β-cyclodextrin) allowed the separation of four pairs of enantiomers. The results are shown in Table 2.

## 3. Discussion

The essential oil yield from fresh plant material was 1.38% (*w*/*w*). This result is similar to those obtained from the essential oil of *Z. leprieurii* fruits (Cameroon), with a yield of 1.1% [31]. Studies conducted in Venezuela on the essential oil of *Zanthoxylum* sp. fruits reported a yield of 0.5% based on fresh plant material, with the oil being pale yellow in color and highly fragrant [32].

In other studies on *Z. armatum*, the essential oil from the seeds yielded 1.2% (Singh et al., 2013). The yield of essential oil can be influenced by environmental conditions such as temperature, precipitation, soil conditions, and altitude [33].

The density of the essential oil from *Z. lepidopteriphilum* was 0.87 g/mL, which is similar to the 0.98 g/mL reported for *Z. purpureum* [34]. The optical activity value of the oil was +1.86. This property is useful for detecting adulterations, such as the addition of synthetic compounds or components from different botanical origins, which can alter the biological or olfactory properties related to the stereochemical nature of the oil [35].

A total of thirty-three compounds were identified, accounting for 99.62% of the essential oil’s composition on the DB-5ms column and 99.30% on the HP-INNOWax column. The most abundant constituents were oxygenated monoterpenes (90.21%) and hydrocarbon monoterpenes (8.50%), with similar values observed across both polar and nonpolar columns. Among the oxygenated monoterpenes, the primary components were *α*-thujone (70.26%) and *β*-thujone (10.78%), with smaller amounts of sabinene and terpinen-4-ol.

The results obtained were compared with those reported in the literature. Research investigations conducted in Colombia on various species of *Zanthoxylum* have primarily reported high concentrations of monoterpenes and sesquiterpenes, which constitute 89% to 99% of the essential oil composition. In *Z. rhoifolium*, monoterpenes account for 80.5%, while in *Z. monophyllum* they represent 71.6%, and in *Z. fagara*, 6.16%. The predominant compounds in the essential oil of *Z. rhoifolium* include β-myrcene, β-phellandrene, and germacrene D. In contrast, *Z. monophyllum* oil is characterized by sabinene, 1,8-cineole, and cis-4-thujanol, while the essential oil from the fruits of *Z. fagara* predominantly contains germacrene D-4-ol, elemol, and α-cineole [8].

In Venezuela, studies on the fruits of a newly identified species of *Zanthoxylum* revealed a high content of oxygenated monoterpenes (59.15%) and hydrocarbon monoterpenes (38.65%) [32]. Similarly, research in China on *Z. acanthopodium* essential oil found 24.24% oxygenated monoterpenes and 13.98% hydrocarbon monoterpenes [36]. Further studies in northeastern China on *Z. schinifolium* reported 94.33% monoterpenoid compounds in the essential oil from the fruit, while the oil from the leaves contained 50.62% monoterpenoids, 4.23% sesquiterpenoids, and the remainder being sesquiterpenes [37].

Research on the volatile oil from *Z. limonella* fruits identified 88.34% hydrocarbon monoterpenes and 8.26% oxygenated monoterpenes, with a smaller percentage of sesquiterpenes. The main constituents were sabinene (42.73%), limonene (39.05%), and terpinen-4-ol (5.40%) [38]. Additionally, the essential oil from the leaves, stems, and seeds of *Z. alatum* was found to contain 41.58% oxygenated monoterpenes and 31.1% hydrocarbon monoterpenes [39].

Enantiomers exhibit the same chemical properties, as their enantiomeric distribution is not typically altered by the distillation process, unlike the overall chemical composition of the oil.

Thujone is a bicyclic monoterpene ketone naturally found in the essential oils of various plants. Biosynthetically, thujone is believed to be produced through an enzyme-mediated reduction of sabinone. The known diastereomers of thujone exhibit different toxicities, with (−)-α-thujone reported to be more toxic than the (+)-β diastereomer. Animal studies have demonstrated that (−)-α-thujone is more potent than (+)-β-thujone. Additionally, (−)-α-thujone has been explored for its potential in treating nausea associated with chemotherapy, radiation therapy, and withdrawal from drug addiction. Regarding health effects, the presence of α-thujone or β-thujone in foods and beverages is regulated in various countries [40,41,42].

## 4. Materials and Methods

### 4.1. General Information

The chemical analysis of *Z. lepidopteriphilum* essential oil (EO) was performed using a gas chromatography–mass spectrometry (GC-MS) system, consisting of an Agilent Technologies 6890 N gas chromatograph equipped with an autoinjector (Model 7683) and coupled to a simple quadrupole mass spectrometry detector (MSD) (Agilent Technologies, Santa Clara, CA, USA). Both qualitative and quantitative analyses were conducted using non-polar- and polar stationary-phase capillary columns from Agilent Technologies. Enantioselective analysis was performed on a chiral stationary-phase column containing 30% diethyl-tert-butyldimethylsilyl-β-cyclodextrin in PS-086, purchased from Mega (Milan, Italy). For all analyses, GC-purity-grade helium (Indura, Guayaquil, Ecuador) was used as the carrier gas. Analytical-grade solvents, a mixture of n-alkanes (C9–C25), and dichloromethane were obtained from Sigma-Aldrich (St. Louis, MO, USA).

### 4.2. Plant Material

The re-collection of the plan material of *Zanthoxylum lepidopteriphilum* was at the fructifying stage on December 2022, in Celica, Loja Province, southern Ecuador (4°05′58″ S y 79°57′08″ W, 2110 m above the sea level, under permission of the Ministry of Environment, Water, and Ecological Transition of Ecuador, with MAATE registry number MAE-DNB-CM-2016-0048). The taxonomical identification was carried out by one of the authors (N.C.), and a botanical specimen is conserved at the herbarium of the Universidad Técnica Particular de Loja with voucher HUTPL 15151. The fresh plant material was steam distilled the same day of collection.

### 4.3. Extraction of Essential Oil

The fruit pericarp (200 g) was subjected to hydro-distillation immediately after harvesting in a Clevenger-type apparatus for four hours [37,38]. The EO collected was dried over anhydrous sodium sulfate and stored in vials protected at 4 °C until further analysis. The procedure was performed three times.

### 4.4. Physical Properties

Physical characterization of *Z. lepidopteriphilum* essential oil was determined by triplicate at 20 °C. A pycnometer of 1 mL and an analytical balance (Radwag AS 310/C/2, ±0.0001 g) were used to determine the density according to standard ANFOR NF T75-111 [43]. Refractive index was measured on a refractometer model ABBE based on standard ANFOR NF 75-112 [44]. The standard ISO 592-1998 was used for optical activity measurement by means of a polarimeter (model AUTOPOL 880 Automatic Saccharimeter, ±0.03, 10 °C–30 °C) [45].

### 4.5. Gas Chromatography–Mass Spectrometry (GC-MS)

The GC-MS analysis of the essential oil composition was performed using an Agilent Chromatograph (6890N series), coupled to a mass spectrometer detector (Agilent series 5973 inert); the spectrometer was controlled by the data system MSD-Chemstation D.01.00 SP1. Two capillary columns were used: a non-polar DB-5ms 5%-phenyl-methylpolyxilosane and polar HP-INNOWax (polyethyleneglycol) Agilent 19091N-133, capillary columns 30 m × 0.25 mm, thicknesses 0.25 µm. The mass spectrometer was operated in electron impact ionization mode at 70 eV, with a mass range of *m*/*z* 40–350 in full scan mode. The ion source temperature was set at 220 °C and the transfer line at 230 °C.

The initial oven temperature was held at 50 °C for 5 min with a ramp of 3 °C/minute until reaching 180 °C and a second ramp of 15 °C/minute until reaching finally the temperature of 230 °C; the injector temperature was 250 °C; the split ratio was adjusted at 20:1; and helium was used as a carrier gas (0.9 mL/minute) in constant flow mode and a mass range of 40–350 *m*/*z*. Essential oil samples were diluted in ciclohexane, ratio 1:100 (*v*/*v*), and 1 μL of the solution was injected.

### 4.6. Gas Chromatography-Coupled Flame Ionization Detector Analysis (GC-FID)

Quantitative analysis of the essential oil was performed on an Agilent Technologies chromatograph (model 6890N series) using a flame ionization detector (FID). The same capillary columns and analytical parameters as those used in the GC-MS measurement were also used in the GC-FID analysis.

### 4.7. Identification and Quantification of Compounds

Identification of the volatile constituents was archived by comparison of their retention indices (RI) with a mass spectrum with retention indices and mass spectra reported in Adams (2009). The experiment retention indices were calculated according to the literature [39] in reference to a homologous series of n-alkanes C9–C20 under identical experimental conditions. Quantification of volatile components was performed using data integration by GC-FID in areas of each peak.

### 4.8. Enantioselective Distribution

Enantioselective analysis of the essential oil was carried out by GC-MS and performed using the same Agilent Technologies instrument described previously. The helium flow was 1 mL/min. The injector was operated in 40:1 split mode at 220 °C. The oven thermal program was as follows: 50 °C, with an initial time of 5 min and a maximum temperature of 230 °C, with an initial temperature ramp of 2 °C/min until reaching 220 °C. The chiral capillary columns used were diethyl tert-butyl silyl-Beta-cyclodextrin (25 m × 0.25 mm, 0.25 µm film thickness).

A homologous series of n-alkanes (C9–C25) was also injected to calculate the linear retention indices of the stereoisomers. The enantiomers were identified based on their mass spectra and elution order, which was determined through the injection of enantiomerically pure standards.

## 5. Conclusions

The EO of *Zanthoxylum lepidopteriphilum* was obtained from the fruits of the plant, with a very high extraction yield of 1.38%. The chemical composition of the EO included 33 compounds, of which *α*-thujone, *β*-thujone, terpinen-4-ol, and sabinene were the most heavily represented. The enantioselective analysis revealed the presence of four pairs of optical isomers, which were (+)-α-pinene, (−) sabinene, (+) α-thujone, and (+)-terpinen-4-ol. 

## Figures and Tables

**Table 1:** Chemical composition of the essential oil from *Zanthoxylum lepidopteriphilum* fruits.

Nº	Compound	DB-5ms			HP-INNOWax		
		LRI^cal^	LRI^ref^ [17]	%	LRI^cal^	LRI^ref^	%
1	*α*-thujene	925	924	0.10 ± 0.01	1019	1020 [18]	0.12 ± 0.01
2	*α*-pinene	931	932	0.23 ± 0.03	1014	1116 [18]	0.26 ± 0.03
3	Sabinene	971	969	3.60 ± 0.30	1117	1112 [18]	4.02 ± 0.34
4	*β*-pinene	976	974	0.22 ± 0.01	1103	1100 [18]	0.22 ± 0.02
5	Myrcene	989	988	0.48 ± 0.03	1163	1161 [19]	0.51 ± 0.04
6	*α*-phellandrene	1005	1002	0.68 ± 0.04	1160	1168 [20]	0.72 ± 0.06
7	*α*-terpinene	1015	1014	0.48 ± 0.05	1175	1166 [18]	0.52 ± 0.06
8	*p*-cymene	1023	1020	0.11 ± 0.01	1268	1269 [20]	0.12 ± 0.02
9	Limonene	1028	1024	1.12 ± 0.07	1195	1194 [19]	0.92 ± 0.07
10	Eucalyptol	1031	1026	0.64 ± 0.02	1202	1202 [21]	0.65 ± 0.03
11	(*E*)-*β*-ocimene	1046	1044	0.42 ± 0.03	1253	1252 [19]	0.42 ± 0.03
12	Terpinene <γ->	1057	1054	0.87 ± 0.09	1242	1238 [19]	0.91 ± 0.10
13	Trans-4-thujanol	1069	1065	0.43 ± 0.06	1464	1458 [20]	0.44 ± 0.05
14	Terpinolene	1084	1086	0.21 ± 0.02	1279	1265 [22]	0.22 ± 0.02
15	Cis-4-thujanol	1101	1098	0.35 ± 0.04	1548	1555 [23]	0.36 ± 0.18
16	*α*-thujone	1107	1101	70.26 ± 1.81	1417	1403 [24]	70.38 ± 1.72
17	*β*-thujone	1118	1112	10.78 ± 1.46	1434	1421 [24]	10.90 ± 1.45
18	Cis-p-2-menthen-1-ol	1124	1118	0.33 ± 0.05	1556	1638 [25]	0.13 ± 0.01
19	Trans-p-2-menthen-1-ol	1142	1136	0.21 ± 0.02	1562	1571 [26]	0.32 ± 0.02
20	Trans-verbenol	1145	1140	0.15 ± 0.01	-	-	-
21	Neo-thujol	1153	1149	0.24 ± 0.03	-	-	-
22	3-thujanol	1169	1164	0.24 ± 0.01	-	-	-
23	Pinocamphone <cis->	1175	1172	1.44 ± 0.08	1536	1565 [26]	1.63 ± 0.09
24	Terpinen-4-ol	1180	1174	4.15 ± 0.52	1601	1602 [19]	4.06 ± 0.49
25	Myrtenol	1192	1194	0.23 ± 0.04	1805	1805 [26]	0.00
26	*γ*-terpineol	1194	1199	0.37 ± 0.02	1628	1685 [27]	0.20 ± 0.02
27	Carvotanacetone	1248	1244	0.25 ± 0.02	1670	1697 [25]	0.15 ± 0.02
28	Thujanol acetate <iso-3->	1269	1267	0.20 ± 0.02	-	-	-
29	Sabinyl acetate <trans->	1288	1289	0.43 ± 0.08	1652	1658 [28]	0.30 ± 0.01
30	Carvacrol	1298	1298	0.14 ± 0.01	-	-	-
31	Myrtenyl acetate	1316	1324	0.28 ± 0.02	-	-	-
32	*α*-terpinolene	-	-	-	1698	1297 [29]	0.42 ± 0.10
33	Camphene	-	-	-	1679	1076 [30]	0.43 ± 0.04
Monoterpenes hydrocarbons				8.50			8.94
Oxygenated monoterpenes				90.21			89.21
Others				0.91			0.30
**TOTAL**				**99.62**			**99.30**

Notes: LRI^cal^: linear retention index calculated; LRI^ref^ [17]: linear retention index of the literature, Adams (2009); IRL^ref.^: linear retention indices references [18] (Morocho et al., 2017); [19] (Valarezo et al., 2013), [20] (El Asbahani et al., 2015); [21] (Mighr et al., 2010); [22] (Noumi et al., 2018); [23] (Tzakou and Loukis, 2009); [24] (Fan et al., 2018); [25] (Başer, Demirci, Kirimer, Satil, and Tümen, 2002); [26] (Maggio et al., 2012); [27] (Hua, Wang, and Lei, 2011); [28] (Tenore et al., 2011); [29] (Pulido, Riveros, and Rodriguez, 2018); [30] (Özek et al., 2014).

**Table 2:** Enantiomeric analysis of the components of *Z. lepidopteriphilum* essential oil.

Compound	RT	LRI^cal^	Enantiomeric Distribution %	e.e. (%)
(*1R*,*5R*)-(+)-α-pinene	9.29	924	50.33	0.65
(*1S*,*5S*)-(−)-α-pinene	9.71	932	49.67	
(*1R*,*5R*)-(+)-sabinene	12.59	986	4.78	90.44
(1S,5S)-(−)-sabinene	13.20	998	95.22	
(1R,4S,5S)-(+)-α-thujone	21.91	1147	96.98	84.40
(*1S*,*4R*,*5R*)-(−)-α-thujone	24.76	1195	8.21	
(4S)-(+)-terpinen-4-ol	28.97	1269	68.78	18.50
(4R)-(−)-terpinen-4-ol	29.07	1270	31.22	

Notes: RT: retention time (minutes); LRI^cal^: linear retention indices calculated in reference to a homologous series of n-alkanes on MEGA-DEX DET beta capillary column; e.e.: enantiomeric excess.

## Data Availability

Data are contained within the article.
